# Dynamic Flying Ant Colony Optimization (DFACO) for Solving the Traveling Salesman Problem

**DOI:** 10.3390/s19081837

**Published:** 2019-04-17

**Authors:** Fadl Dahan, Khalil El Hindi, Hassan Mathkour, Hussien AlSalman

**Affiliations:** 1 Department of Information System, College of Computer Engineering and Science, Prince Sattam Bin Abdulaziz University, Al Kharj 11942, Saudi Arabia; f.naji@psau.edu.sa; 2 Department of Computer Science, College of Computer and Information Sciences, King Saud University, Riyadh 11543, Saudi Arabia; Mathkour@KSU.EDU.SA (H.M.); halsalman@KSU.EDU.SA (H.A.)

**Keywords:** traveling salesman problem (TSP), ant colony optimization (ACO), flying ant colony optimization (FACO), dynamic flying ant colony optimization (DFACO)

## Abstract

This paper presents an adaptation of the flying ant colony optimization (FACO) algorithm to solve the traveling salesman problem (TSP). This new modification is called dynamic flying ant colony optimization (DFACO). FACO was originally proposed to solve the quality of service (QoS)-aware web service selection problem. Many researchers have addressed the TSP, but most solutions could not avoid the stagnation problem. In FACO, a flying ant deposits a pheromone by injecting it from a distance; therefore, not only the nodes on the path but also the neighboring nodes receive the pheromone. The amount of pheromone a neighboring node receives is inversely proportional to the distance between it and the node on the path. In this work, we modified the FACO algorithm to make it suitable for TSP in several ways. For example, the number of neighboring nodes that received pheromones varied depending on the quality of the solution compared to the rest of the solutions. This helped to balance the exploration and exploitation strategies. We also embedded the 3-Opt algorithm to improve the solution by mitigating the effect of the stagnation problem. Moreover, the colony contained a combination of regular and flying ants. These modifications aim to help the DFACO algorithm obtain better solutions in less processing time and avoid getting stuck in local minima. This work compared DFACO with (1) ACO and five different methods using 24 TSP datasets and (2) parallel ACO (PACO)-3Opt using 22 TSP datasets. The empirical results showed that DFACO achieved the best results compared with ACO and the five different methods for most of the datasets (23 out of 24) in terms of the quality of the solutions. Further, it achieved better results compared with PACO-3Opt for most of the datasets (20 out of 21) in terms of solution quality and execution time.

## 1. Introduction

The traveling salesman problem (TSP) [1] involves finding the shortest tour distance for a salesperson who wants to visit each city in a group of fully connected cities exactly once. TSP is a discrete optimization problem. It is a classic example of a category of computing problems known as NP-hard problems [2,3]. Although there are simple algorithms for solving these problems, these algorithms require exponential time, which makes them impractical for solving large-size problems. Hence, metaheuristic optimization algorithms are usually applied to find good solutions, although these solutions may not be optimal.

The TSP problem can be used for modeling several wireless sensor network (WSN) problems [4]. In a WSN, the sensors are located in a sensing field to collect data, and send these data to the source node wirelessly. There are two ways to increase the lifetime of the sensors: first, by reducing the size and number of data [5,6], and second, by reducing the cost of transferring the data [4]. For example, a good solution to the TSP problem can also be considered an efficient diffusion method for reducing the transferring cost.

Many methods, including heuristic or hybrid, have been proposed for solving the TSP, but most of them were unable to avoid the stagnation problem, or they may have obtained good solutions but took a long execution time to do so [7]. In this work, we enhanced the ant colony optimization (ACO) algorithm based on imaginary ants that can fly. These ants deposit their pheromones with neighboring nodes while flying by injecting them from a distance. This allows not only the nodes on a good path to receive some pheromones but also the neighboring nodes. The algorithm also makes use of the 3-Opt algorithm to help avoid reaching a local minimum.

The main contributions of this work on the flying ACO (FACO) algorithm are as follows: (1) proposing a dynamic neighboring selection mechanism to balance between exploration and exploitation, (2) reducing the execution time of FACO by making flying ants equal to half the ants, and (3) adapting the flying process to work with the TSP problem.

The main contributions of this work in general are as follows: (1) obtaining better-quality solutions, (2) significantly reducing the execution time, and (3) avoiding getting stuck at a local minimum.

The paper is organized as follows: in Section 2, we discuss related works; Section 3 presents the proposed enhancement of the ACO algorithm; Section 4 shows the experimental results; and the conclusion is shown in Section 5.

## 2. Related Work

This section reviews some important and more recent works in this area.

### 2.1. Metaheuristic Solutions

The TSP has been widely used as a benchmark problem to evaluate many metaheuristic and nature-inspired algorithms. Chen and Chien [8] presented a hybrid method using simulated genetic annealing, ACO, and particle swarm optimization. Each algorithm performs a specific task, where ACO generates the initial solutions for the genetic simulated annealing algorithm, which searches for better solutions based on the initial solutions. Then, the better solutions are used to update the pheromone trails. Finally, the particle swarm optimization algorithm exchanges the pheromone information after a predefined number of cycles. Deng et al. [9] presented a hybrid method that combined a genetic algorithm and ACO. They used a multipopulation strategy to enhance the local search. In addition, they used chaotic optimization to avoid the ACO slow convergence problem. They controlled the trade-off between exploration and exploitation by using an adaptive control strategy to distribute the pheromones uniformly. Eskandari et al. [10] proposed a local solution enhancement for ACO based on mutation operators. The local solution is mutated to generate a new solution and keeps it if it is better than the original solution. The mutation operators include swap, insertion, and reversion. A comparison between ACO and cuckoo search (CS) algorithms was conducted in [11] for solving the TSP. In this comparison, only five city plans were used. Mavrovouniotis et al. [12] used local search operators to support the ACO algorithm. This new method was used for dynamic TSP. The best solution from ACO is passed to local search operators for removing and inserting cities to generate a new solution. Alves et al. [13] introduced an adapted ACO algorithm based on social interaction called social interaction ant colony optimization (SIACO). The social interaction was introduced to enhance pheromone deposition. Han et al. [14] proposed a niching ant colony system (NACS) algorithm. This algorithm enhances the ACO algorithm in two ways: it applies a niching strategy and uses multiple pheromone deposition. Pintea et al. [15] introduced an enhancement for ACO based on clustering, where the cities are divided into clusters and ACO is used to find the minimum cost for each cluster. Xiao et al. [16] proposed a multistage ACO algorithm that reduces the initial pheromone concentration based on the nearest-neighbor method. Then, the mean cross-evolution strategy is used to enhance the solution space. Zhou et al. [17] proposed an ACO algorithm for large-scale problems that utilizes graphics processing units.

The 3-Opt algorithm is widely used to enhance the local search of metaheuristic algorithms. Mahi et al. [18] presented a hybrid algorithm using particle swarm optimization and ACO. The particle optimizes the α and β parameters, which affect the performance of ACO. The 3-Opt algorithm is then used to avoid the stagnation problem. Gülcü et al. [7] introduced a parallel cooperative method, which is a hybridization of the parallel ACO (PACO) and the 3-Opt algorithm. The proposed algorithm is named as PACO-3Opt and it uses a parallel set of colonies. These multiple colonies have a master–slave paradigm. The 3-Opt algorithm is used by these colonies based on a predefined number of iterations. Khan et al. [19] used 2-Opt and 3-Opt with an artificial bee colony (ABC) algorithm. Also, they created a new different path by combing swap sequences with ABC.

Ouaarab et al. [20] proposed a discretized version of the CS algorithm and also added a new cuckoo category. This new category aims to manage the exploration and exploitation by using Lévy flights and multiple searching methods. Osaba et al. [21] presented a discrete version of the bat algorithm where each bat moves based on the best bat. If a bat is located far from the best bat, then the movement will be large, but if it is close to the best bat, the step will be small. Choong et al. [22] introduced a hybrid algorithm of the ABC algorithm and modified choice function. The modified choice function is used to regulate the neighborhood selection of employed and onlooker bees.

Many works in the literature present improvements on existing algorithms by suggesting methods to control the tradeoff between exploitation and exploration, which are the two main steps that form the basis of many metaheuristic approaches and nature-inspired algorithms. In exploitation, the accumulated knowledge about the search space is used to guide the search, while in exploration, risk is taken to explore the unfamiliar region of the search space, in the hope that this region may contain a solution better than the known solutions [23].

Researchers usually use hybrid methods to merge different algorithms’ capabilities; however, new methods can become too complex and sometimes even incomprehensible. By contrast, we aimed in this work to enhance the ACO algorithm by adding extra procedures while keeping the method understandable and easy to use.

### 2.2. Opt Algorithm

The 3-Opt algorithm was introduced to solve the TSP. It exchanges three edges from the old tour by another three edges to produce a new tour [24] and retains the new tour if it is better. The process is repeated until no further improvement is found. 3-Opt is a local search algorithm; therefore, it is used to help ACO avoid local minima [7] by optimizing the solutions locally [25].

**Figure 1:**
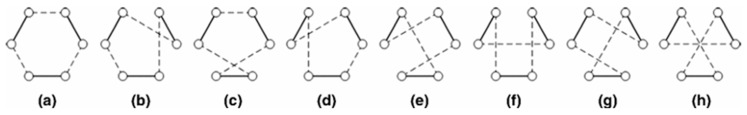
3-Opt possible combinations.

There are $\left(\begin{array}{c} n\\ 3 \end{array}\right)$. possible combinations to replace three edges from the tour with n cities [26]. For instance, from three edges, we can obtain eight possible combinations, as shown in Figure 1 where (a) to (h) represent these eight combinations [27].

The experiments reported in [26,28] show that combining the 3-Opt algorithm with other metaheuristic algorithms improves the solutions found by these algorithms. This is because the 3-Opt algorithm mitigates the effect of local minima [28].

### 2.3. Ant Colony Optimization

ACO was inspired by the way real ants forage for food. Initially, real ants forage for food randomly, depositing a chemical substances called pheromones on their paths. The path between the colony and the nearest food source tends to receive more pheromones, which attracts more ants to follow the same path. Once the food source is exhausted, the ants abandon the path and the pheromones evaporate, forcing the ants to start searching for another food source randomly.

The ACO algorithm [28,29,30] simulates the foraging behavior of real ants. It initializes all ants randomly, and each ant searches for a potential solution. In addition, ACO assigns an amount of pheromone to each edge of the solution path that is proportional to the quality of the solution.

In each iteration, each ant moves to unvisited nodes in order to construct a potential solution. The next node to visit is selected according to a probability distribution that favors the nodes with large amounts of pheromones ($\tau_{ij}$). ACO also takes into account a local heuristic function ($\eta_{ij}$). Equation (1) shows the solution generation formula which computes the probability of selecting the edge from nodes *i* to j:

(1) $$P_{ij}^{k}\left(t\right)=\frac{{\left[\tau_{ij}\right]}^{\alpha }{\left[\eta_{ij}\right]}^{\beta }}{\sum_{l\in N_{i}^{k}}{\left[\tau_{il}\right]}^{\alpha }{\left[\eta_{il}\right]}^{\beta }}\;if\;j\in N_{i}^{k}$$

where *α* and *β* are coefficient parameters that determine the importance of the pheromone value ($\tau_{ij}$) and the value of the local heuristic ($\eta_{ij}$). The local heuristic $\eta_{ij}$ is problem dependent. For the TSP, it is defined as 1 divided by the length of the edge between nodes i and j. *N_i_^k^* is the list of unvisited nodes from node *i* by ant *k*.

The ants update the pheromones locally using Equation (2). This update is called the local pheromone update:

(2) $$\tau_{ij}\left(t+1\right)=\left(1-\rho \right)\tau_{ij}\left(t\right)+{\rho \tau }_{0}$$

where *τ*_0_ is the initial pheromone value, and *ρ* is the evaporation rate.

ACO selects the best solution greedily. The best ant updates the pheromone trails on its path using Equation (3). This process is called the global pheromone update:

(3) $$\tau_{ij}\left(t+1\right)=\left(1-\rho \right)\tau_{ij}\left(t\right)+\rho \Delta \tau_{ij}\left(t\right)$$

$\Delta \tau_{ij}\left(t\right)$ is defined as

(4) Δτij(t)={1Lgb(t) if arc(i,j)∈the best tour0  otherwise

where *L^gb^*(*t*) is the tour length for the best ant.

**Figure 2:**
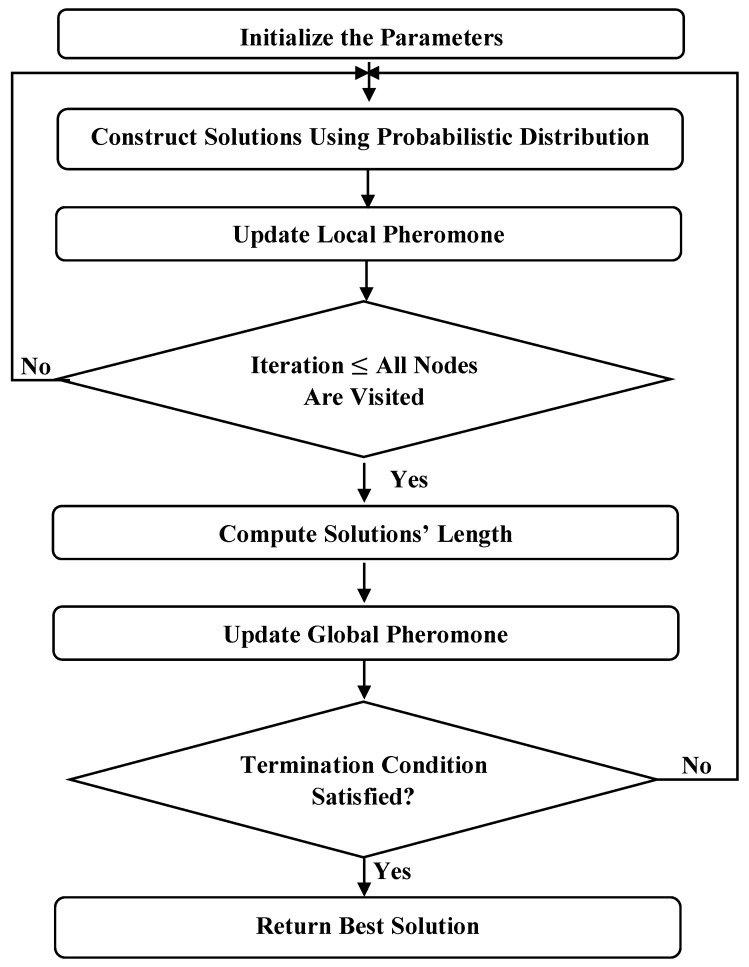
Ant colony optimization (ACO) flowchart.

These ACO algorithm processes are illustrated in Figure 2 [31]. The ACO algorithm suffers from a stagnation problem [32] because the amounts of pheromone are accumulated on the explored paths, and as a result, the chances of exploring other paths decrease. The FACO algorithm, discussed next, addresses this issue.

### 2.4. Flying Ant Colony Optimization

In [33], a flying ant colony algorithm was proposed to solve the quality of service (QoS)-aware web service composition problem. Web service composition involves selecting the best combination of web services, where each service is selected from a set of candidate services that fulfill a certain task. The solutions are evaluated according to a set of QoS properties, such as reliability, cost, response time, and availability.

The algorithm assumes that, in addition to walking normal ants, there are also flying ants. Flying ants inject their pheromones from a distance, so that not only the nodes on the path receive some pheromones but also their neighboring nodes. The amount of pheromone a neighboring node receives is inversely proportional to the distance between it and the node on the path. This makes the ants more likely to explore these nodes during future iterations, which encourages exploration.

Since determining the nearest nodes might be an expensive iteration in terms of execution time, we only considered the ant that finds the best solution as a flying ant in each iteration. The rest of the ants are dealt with in the usual way. The flying ant then determines the nearest neighboring nodes (web services) by using Equation (5) to calculate the distance between two web services x and y. Equation (5) considers two web services that are similar (close to each other) if they have similar QoS properties:

(5) $$d_{\left(x,y\right)}=\sqrt{{\left(C_{x}-C_{y}\right)}^{2}+{\left(RT_{x}-RT_{y}\right)}^{2}+{\left(A_{x}-A_{y}\right)}^{2}+{\left(R_{x}-R_{y}\right)}^{2}}$$

where *C*, *RT*, *A*, and *R* are the cost, response time, availability, and reliability, respectively; *x* is the best web service obtained by the best ant in task *t_i+_*_1_; *y* is one of the neighboring web services to *x* in task *t_i+_*_1_; and *x ≠ y*.

The nearest neighbors receive an amount of pheromone that is inversely proportional to their distance from the best node. Equation (6) describes how much pheromone a node receives:

(6) $$\tau_{\left(i+1,x^{\prime }\right)\left(i+1,l\right)}\left(t+1\right)=\tau_{\left(i+1,x^{\prime }\right)\left(i,l\right)}\left(t\right)+\left(\frac{\tau_{\left(i+1,x\right)\left(i+1,x^{\prime }\right)}\left(t+1\right)}{\left(1+\sqrt{d^{\prime }\left(\eta_{\left(i+1,x^{\prime }\right)\left(i+1,l\right)}\right)}\right)}\right)$$

where l is the neighbor’s number where l∈[1,NS]. *NS* is the number of neighbors. $\tau_{\left(i,x\right)\left(i+1,x^{\prime }\right)}\left(t+1\right)$ represents the pheromone trails from the global pheromone update. $d^{\prime }\left(\eta_{\left(i,x^{\prime }\right)\left(i,l\right)}\right)$ is the normalized distance between web services in task *i* + 1 and its neighbor web service *l* in the same task.

The distance (local heuristic) $\eta_{ij}$. is normalized according to Equation (7):

(7) $$d^{\prime }\left(\eta_{\left(i+1,x^{\prime }\right)\left(i+1,l\right)}\right)=\frac{\eta_{\left(i+1,x^{\prime }\right)\left(i+1,l\right)}}{\sum_{q=1}^{NS}\eta_{\left(i+1,x^{\prime }\right)\left(i+1,q\right)}}$$

**Figure 3:**
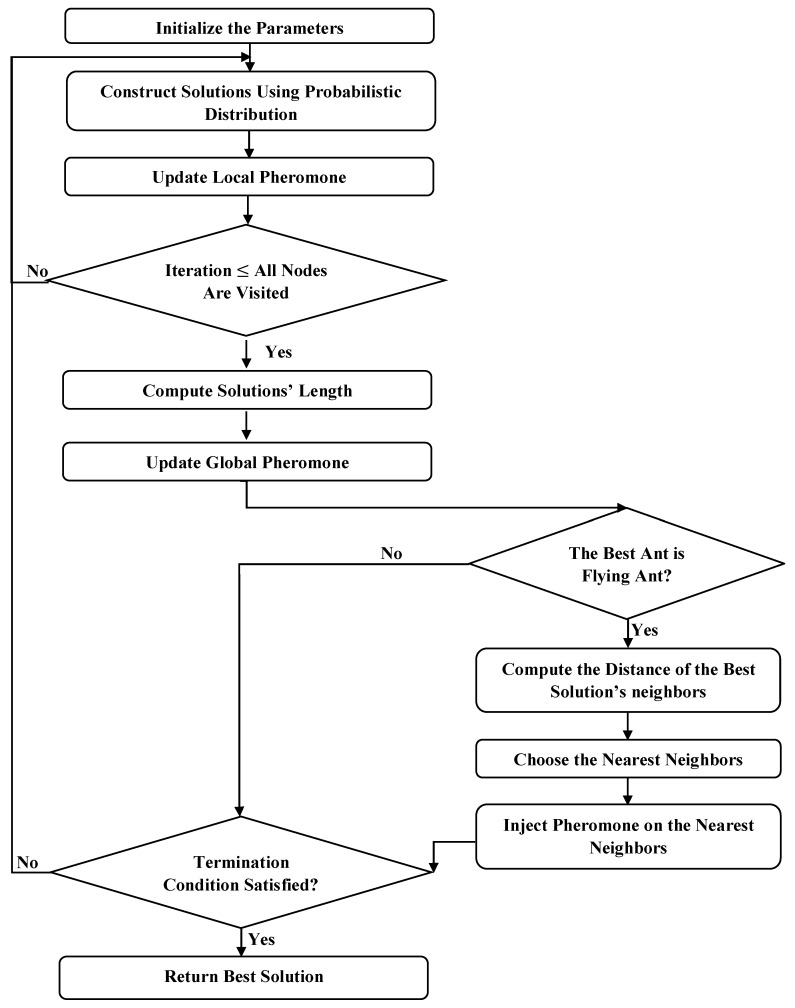
Flying ACO (FACO) flowchart.

Figure 3 illustrates the process of the FACO algorithm [33], which was added to the process of ACO.

## 3. Dynamic Flying Ant Colony Optimization (DFACO) Algorithm

Many methods, including heuristic or hybrid, have been proposed for solving the TSP, but most of them cannot avoid the stagnation problem, or they may obtain solutions but take a long execution time [7]. In this work, we proposed an enhanced ACO algorithm that finds better solutions in less computation time and a robustness mechanism to avoid the stagnation problem.

In this section, we present a modification of the FACO algorithm to make it suitable for the TSP problem in the following ways.

First, the number of neighboring nodes in FACO were static based on the experiments. However, the number of neighbors in DFACO that may be injected with pheromones was dynamic. The number of neighboring nodes varied in each iteration depending on the quality of the best solution reached so far compared to the other solutions. The number of neighbors was determined based on two cases: (1) If the best solution is slightly better than the other solutions, then the number of neighbors should be large to obtain more neighbors. This encourages exploration in future iterations. (2) If the best solution is considerably better than the other solutions, then the number of neighbors should be small to encourage exploitation in future iterations.

The number of nearest neighbors, *NS*, was determined according to the following formula:

(8) $$NS=\left(\frac{L^{gb}\left(t\right)}{\sum_{k\in S}L^{gl}\left(t\right)}\right)\times N$$

where *L^gb^*(*t*) is the tour length of the global best tour at time *t*, *L^kl^*(*t*) is the tour length of ant *k* at time *t*, *S* is the number of ants, and *N* is the number of cities.

The second modification aimed to reduce the execution time. If all ants were flying ants (such as in FACO), we would have to determine many neighbors for each node on the best path, which may substantially increase the execution time. Therefore, here, only 50% of the ants were flying ants and the rest were normal walking ants.

The third modification aimed to encourage exploration at early stages and exploitation at later stages. This modification is similar to the FACO algorithm, but we modified the process to adapt it to the TSP problem. The intuition behind this is that at early stages, we have no idea about the location of the best solution in the search space; and therefore, ants should be encouraged to explore the search space. On the other hand, at later iterations, the region that contains the best solution is more likely to have been located, and therefore, exploitation should be encouraged. This was achieved by injecting pheromones at farther neighbors during early iterations, while at later iterations, we injected the pheromones at only the nearest neighbors. Equation (6) was used to determine the amount of pheromone for each neighbor.

As a final modification, we embedded the 3-Opt algorithm in FACO to reduce the chances of getting stuck at a local minimum.

**Figure 4:**
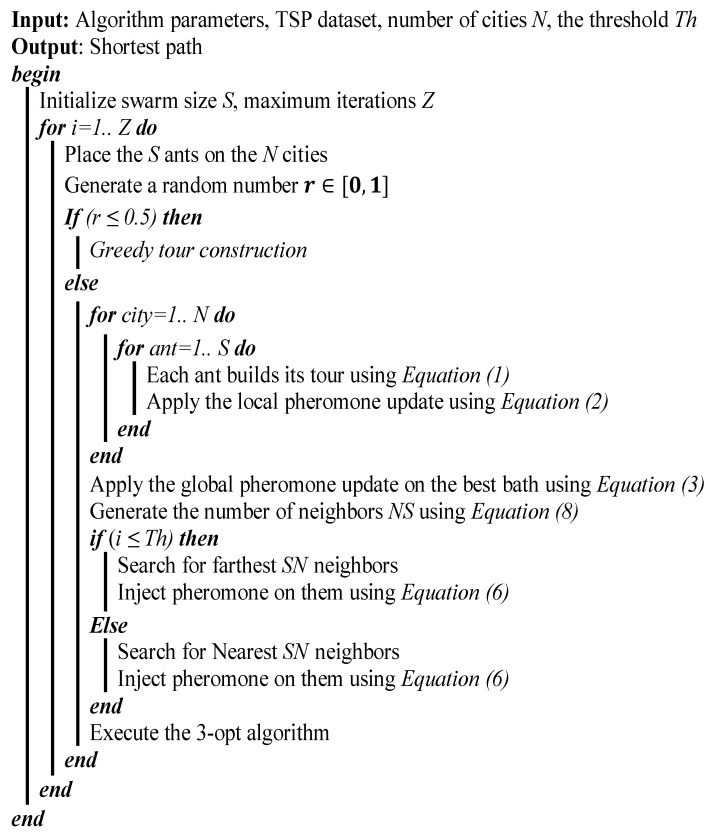
The dynamic FACO (DFACO) algorithm.

**Figure 5:**
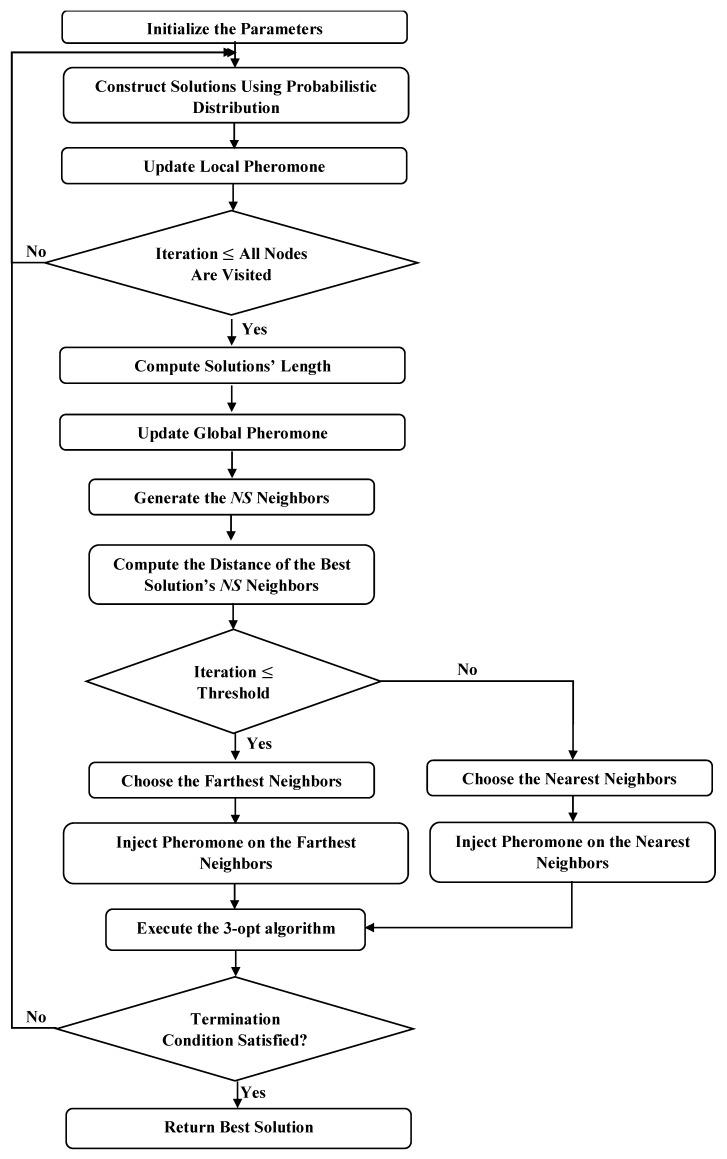
DFACO flowchart.

Figure 4 shows the complete algorithm in detail, and Figure 5 illustrates the process of the DFACO algorithm.

## 4. Experimental Results

We conducted empirical experiments using TSP datasets from TSPLIB [34] to test the performance of the proposed algorithm. We performed three comparisons. First, we compared DFACO combined with the 3-Opt algorithm with ACO combined with the 3-Opt algorithm using 24 datasets. Second, we compared DFACO performance with PACO-3Opt [7] in detail using 21 datasets. Third, we compared DFACO performance with five more recent methods [8,9,18,20,21] using 24 datasets.

We implemented DFACO in Java and used the ACOTSPJava (http://adibaba.github.io/ACOTSPJava/) implementation of ACO.

We compared the methods with respect to the average of the best solutions for all runs (Mean), the standard deviation (SD), and the best solution for each run (Best). We also compared DFACO, PACO-3Opt, and ACO with respect to execution time in seconds.

Table 1 lists the parameter values of both algorithms, which were empirically determined. These values were also used to compare DFACO to all of the other methods.

Both ACO and DFACO were run for 100 iterations (Z) and each dataset was used in 30 independent experiments. Table 2 lists the comparison results for DFACO and ACO. The first column shows the name of the TSP datasets. The second column shows the best-known solution (BKS) as reported on the TSPLIB website (http://comopt.ifi.uni-heidelberg.de/software/TSPLIB95/STSP.html). Bold font indicates the best results.

Table 2 indicates that DFACO was able to find the BKS in all runs for 16 datasets with zero SD, while ACO was able to find the BKS in all runs for 15 datasets with zero SD. However, the proposed method obtained the BKS in all runs faster than ACO for four datasets (bier127, ch130, kroB150, and kroA200), while ACO obtained the BKS faster than DFACO for two datasets (eil101 and ch150).

**Figure 6:**
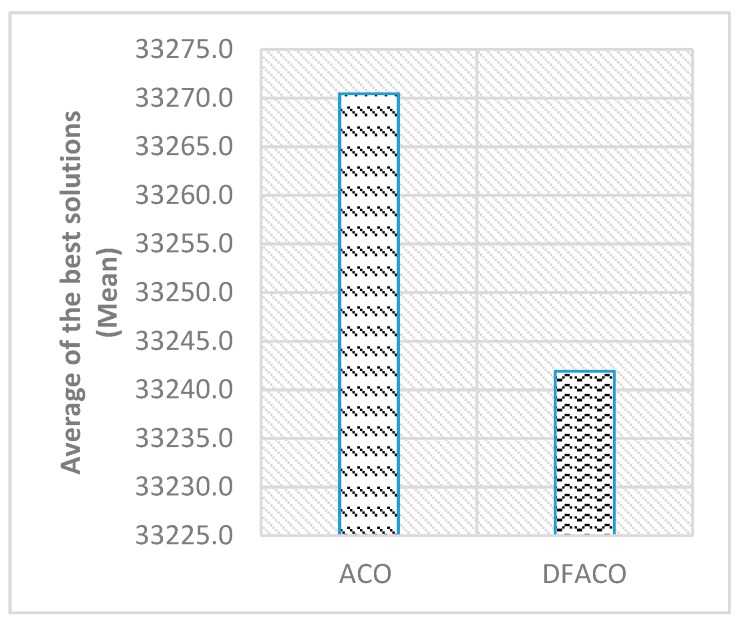
DFACO and ACO comparison in terms of Mean as shown in Table 2.

**Figure 7:**
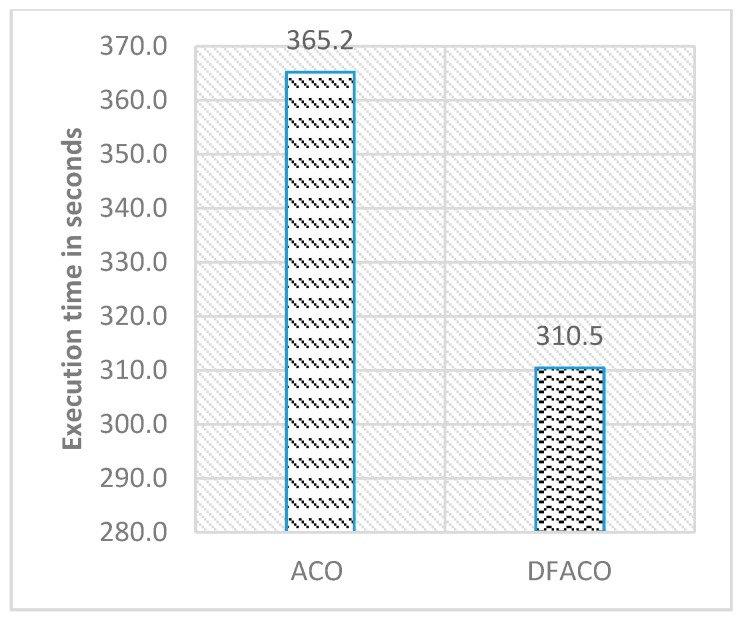
DFACO and ACO comparison in terms of execution time in seconds as shown in Table 2.

Figure 6 shows the average of the best solutions, while Figure 7 presents the execution time in seconds for all datasets in the table. As can be seen in Figure 6, DFACO was better than ACO in terms of the average of the best solutions and obtains a shorter distance on average for all datasets. Also, DFACO was faster than ACO by 54.7 s for all datasets.

For the remaining eight datasets, DFACO found the best Mean solution for seven datasets, while ACO found the best Mean solution for one dataset (fl1400). These results were found to be statistically significant according to the Wilcoxon signed-rank test, with *N* = 8 and *p* ≤ 0.05. A *t*-test was used to see if the results were statistically significant in the 30 independent runs for each one of the eight datasets. The results indicate that the proposed method’s results were statistically significant for four out of eight datasets, namely, for the datasets rat575, rat783, rl1323, and d1655. We did not perform a *t*-test for the remaining 16 datasets because both algorithms achieved the BKS.

For the second set of comparisons, we followed the comparison method of PACO-3Opt [7]. In the PACO-3Opt experiments, the TSP datasets were divided based on the problem size into small-scale and large-scale datasets (the size of the large-scale datasets was between 400 and 600). The small-size datasets used 10 TSP datasets (shown in Table 3), and for the large size, 11 TSP datasets were used (shown in Table 4). Table 3 presents the experimental results of comparing DFACO with PACO-3Opt for small-scale TSP instances. The table shows the best, worst, and average values of both algorithms for comparison. Boldface text indicates the better results for both algorithms. The results reveal that the DFACO obtained the optimum distances for all datasets in terms of best, worst, and average values. Meanwhile, PACO-3Opt obtained the optimum distances for only six datasets, one dataset, and one dataset in terms of best, worst, and average, respectively. With regard to execution time, Table 3 shows that DFACO significantly reduced the execution time and obtained better results faster than PACO-3Opt for all TSP instances.

Table 4 presents the results of comparing DFACO with PACO-3Opt for large-scale TSP instances. It also shows the best, worst, and average values of both algorithms for comparison. Boldface text indicates the better results for both algorithms. The results reveal that DFACO obtained better distances for all datasets in terms of best, worst, and average values, except for rat783. With regard to execution time, Table 4 shows that DFACO significantly reduced the execution time and obtained better results faster than PACO-3Opt for all TSP instances except for rat783, where DFACO was faster than PACO-3Opt but did not obtain better results.

Table 5 shows the third type of comparison between DFACO and five recent methods [5,6,7,8,9]. In this set of experiments, we used 24 TSP datasets and 30 independent runs.

The table reveals that the proposed algorithm achieved the best results for all datasets except one (rat783), for which Deng’s method [9] achieved the best result. Also, DFACO found the BKS for 18 datasets, and for one dataset (rat575), it found an even better solution than the BKS.

Figure 8, Figure 9, Figure 10, Figure 11, Figure 12, Figure 13, Figure 14, Figure 15, Figure 16, Figure 17, Figure 18, Figure 19, Figure 20, Figure 21, Figure 22, Figure 23, Figure 24, Figure 25, Figure 26, Figure 27, Figure 28, Figure 29, Figure 30 and Figure 31 show the average of the best solutions for each TSP instance obtained by different algorithms. These figures visualize the results of the 24 datasets shown in Table 5. From these figures, it is clear that DFACO’s performance was better than that of the other algorithms for most datasets except rat783.

Table 6 compares DFACO with five recent methods with respect to the percentage deviation of the average solution to the BKS value (*PDav*) and the percentage deviation of the best solution to the BKS value (*PDbest*) in the experimental results. *PDav* and *PDbest* were calculated using Equations (9) and (10), respectively:

(9) $$PDav=\frac{Mean-BKS}{BKS}\times 100,$$

(10) $$PDbest=\frac{Best-BKS}{BKS}\times 100.$$

The results revealed that the values of *PDav* and *PDbest* for DFACO were better than those for the other methods on all datasets except one (rat783), for which Deng’s method [9] was better.

## 5. Conclusions

This paper proposed a modified FACO algorithm for the TSP. We modified FACO in several ways to reduce the execution time and to better balance exploration and exploitation. For example, the number of neighboring nodes receiving pheromones varied depending on the quality of the solution compared to the other solutions. This helped to balance the exploration and exploitation strategies. We also embedded the 3-Opt algorithm to improve the solution by mitigating the effect of the stagnation problem. Moreover, the colony contained a combination of normal and flying ants. These modifications aimed to achieve better solutions with less processing time and to avoid getting stuck in local minima.

DFACO was compared with (1) ACO and five recent methods for the TSP [8,9,18,20,21] using 24 TSP datasets and (2) PACO-3Opt using 22 TSP datasets. Our empirical results showed that DFACO achieved the best results compared to ACO and the five different methods for most datasets (23 out of 24) in terms of solution quality. Also, DFACO achieved the best results compared with PACO-3Opt for most datasets (20 out of 21) in terms of solution quality and the execution time. Furthermore, for one dataset, DFACO achieved a better solution than the best-known solution.

## Figures and Tables

**Figure 8:**
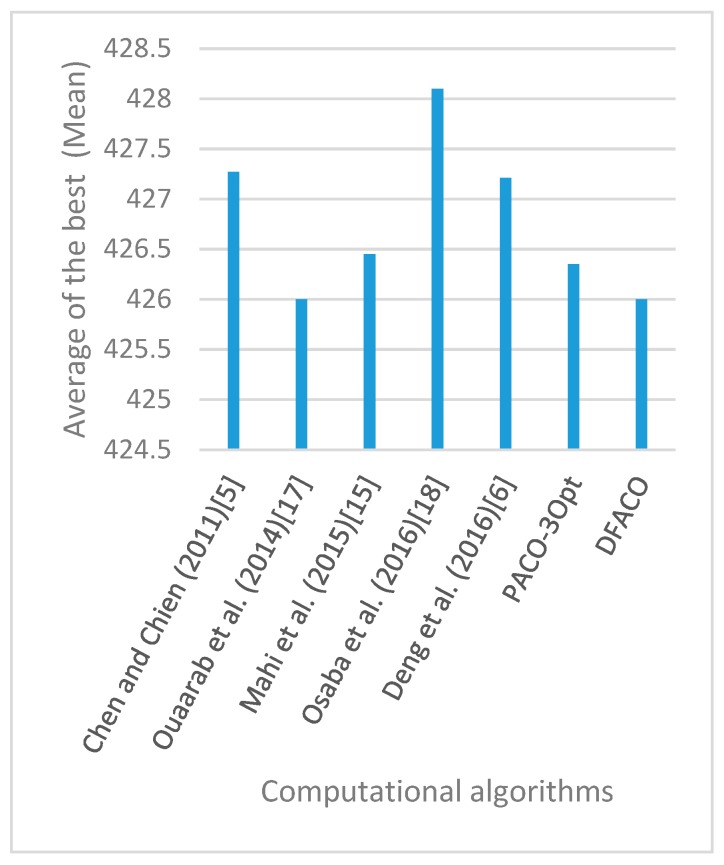
Average of best solutions obtained for instance eil51 by all algorithms.

**Figure 9:**
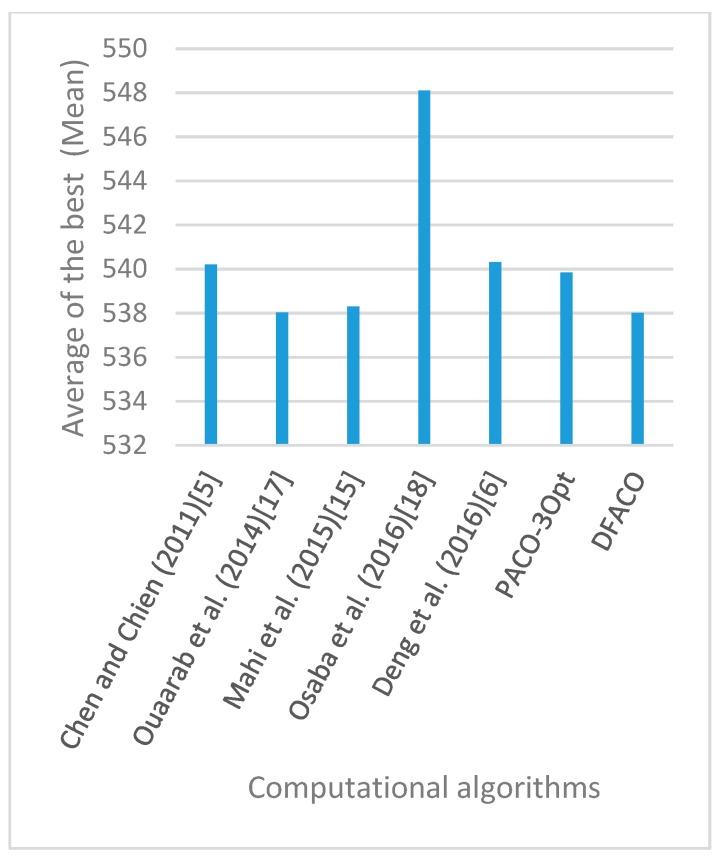
Average of best solutions obtained for instance eil76 by all algorithms.

**Figure 10:**
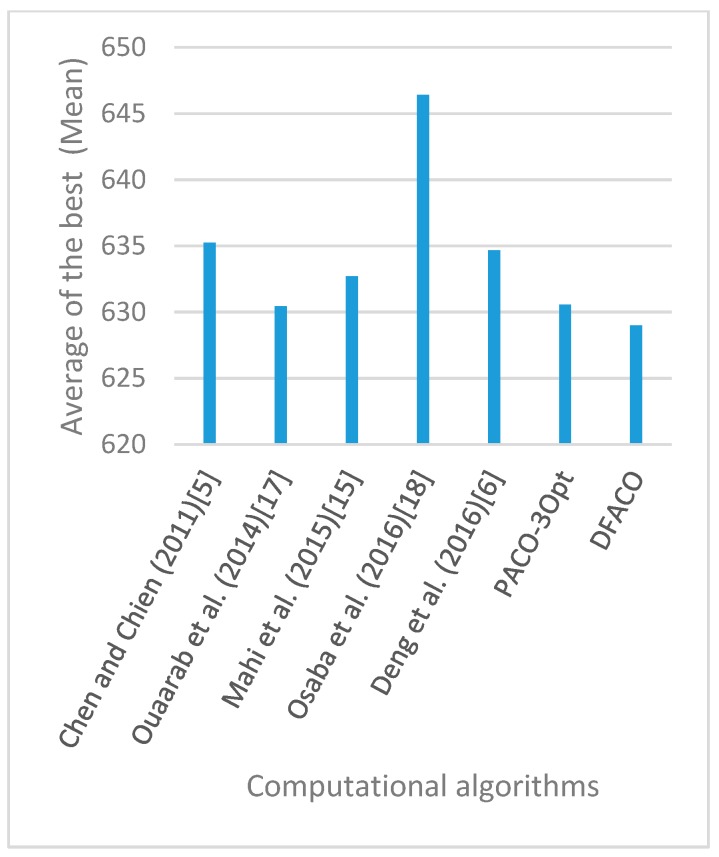
Average of best solutions obtained for instance eil101 by all algorithms.

**Figure 11:**
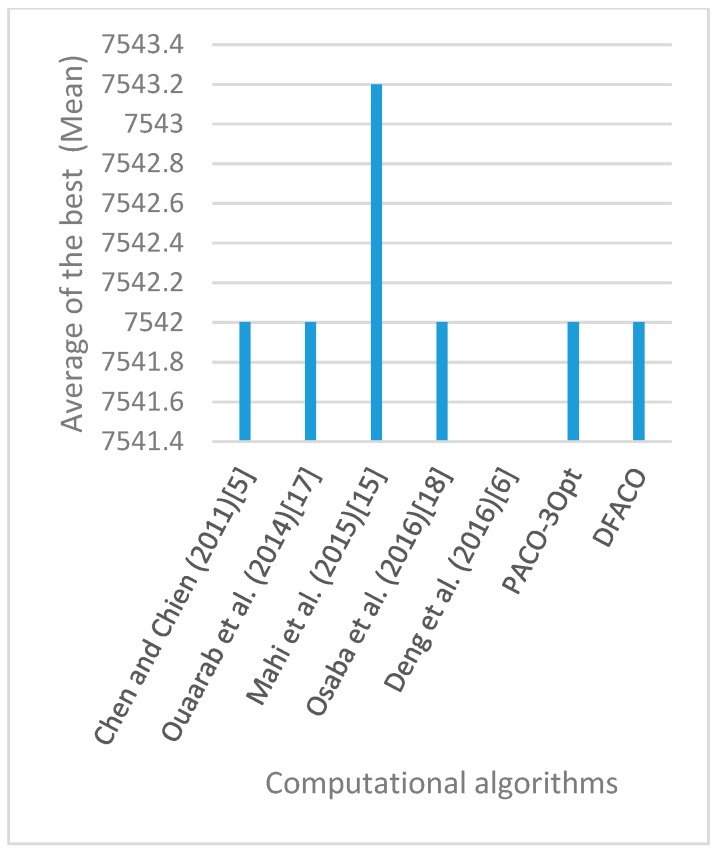
Average of best solutions obtained for instance berlin52 by all algorithms.

**Figure 12:**
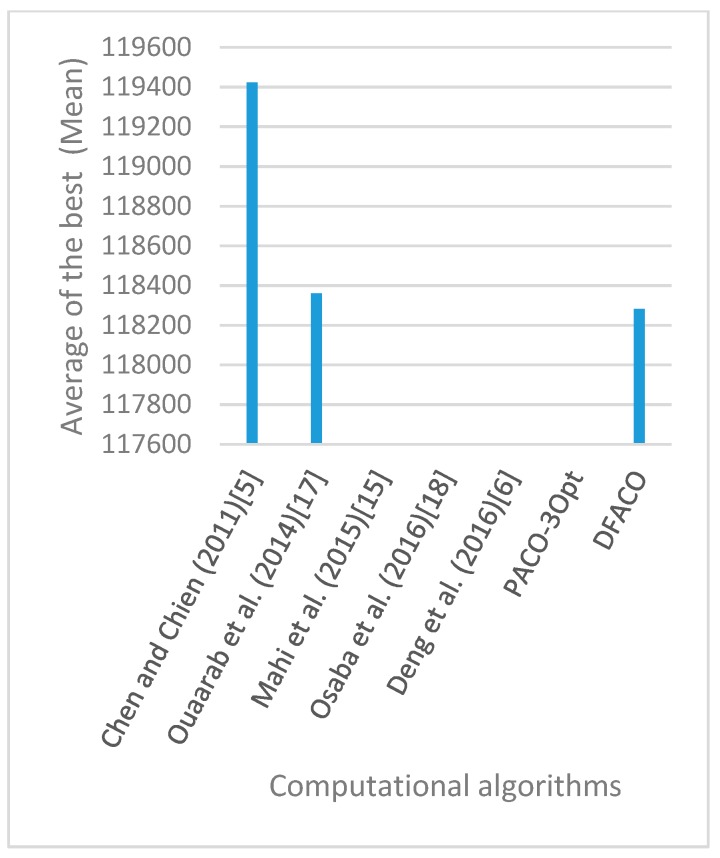
Average of best solutions obtained for instance bier127 by all algorithms.

**Figure 13:**
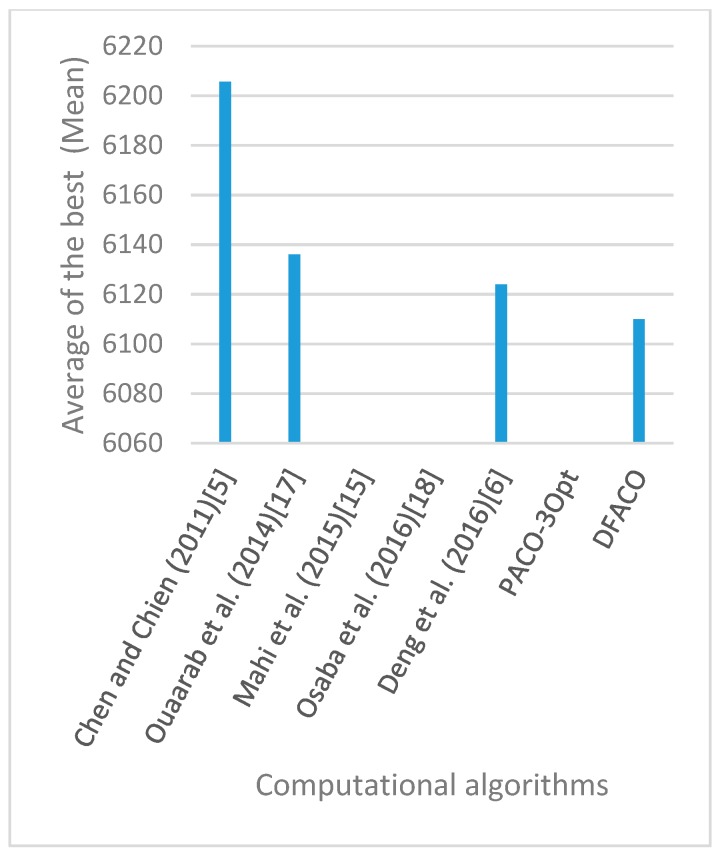
Average of best solutions obtained for instance ch130 by all algorithms.

**Figure 14:**
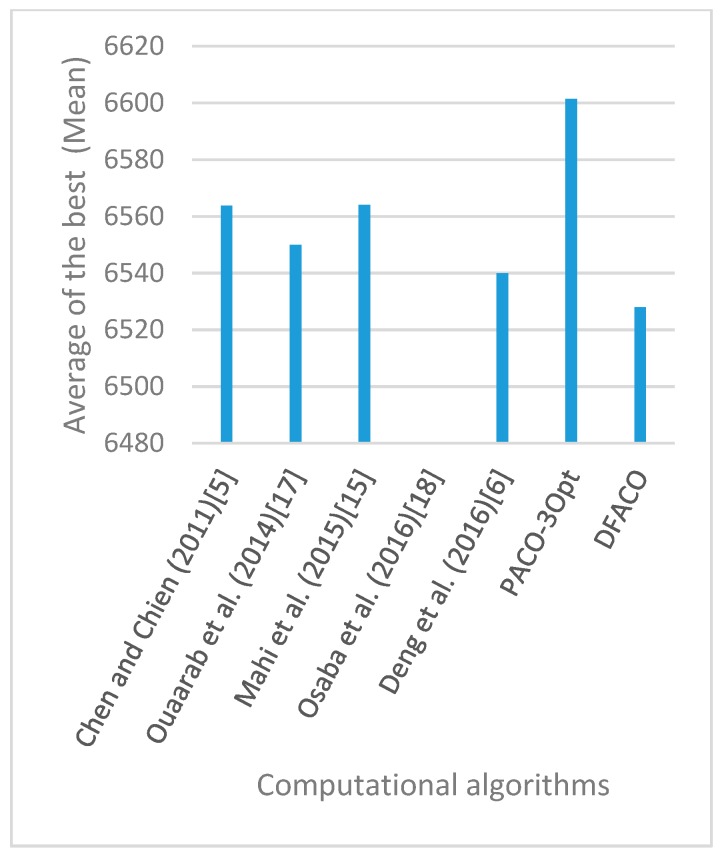
Average of best solutions obtained for instance ch150 by all algorithms.

**Figure 15:**
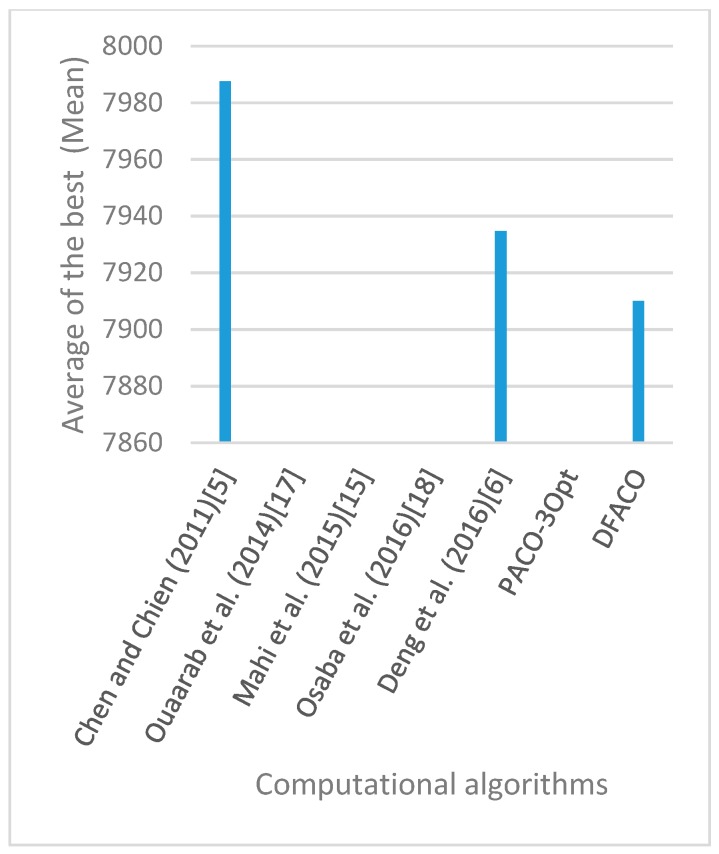
Average of best solutions obtained for instance rd100 by all algorithms.

**Figure 16:**
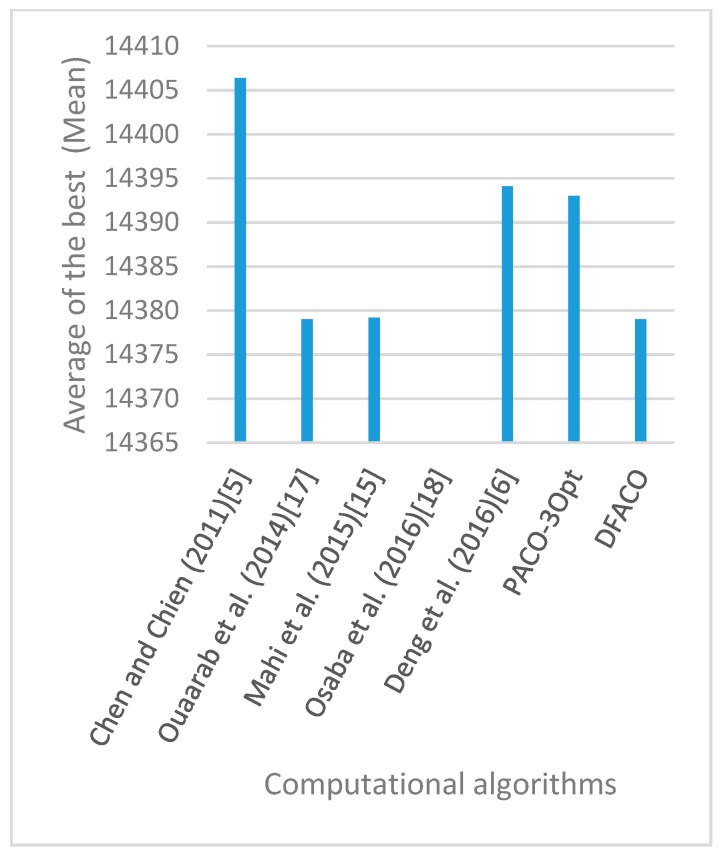
Average of best solutions obtained for instance lin105 by all algorithms.

**Figure 17:**
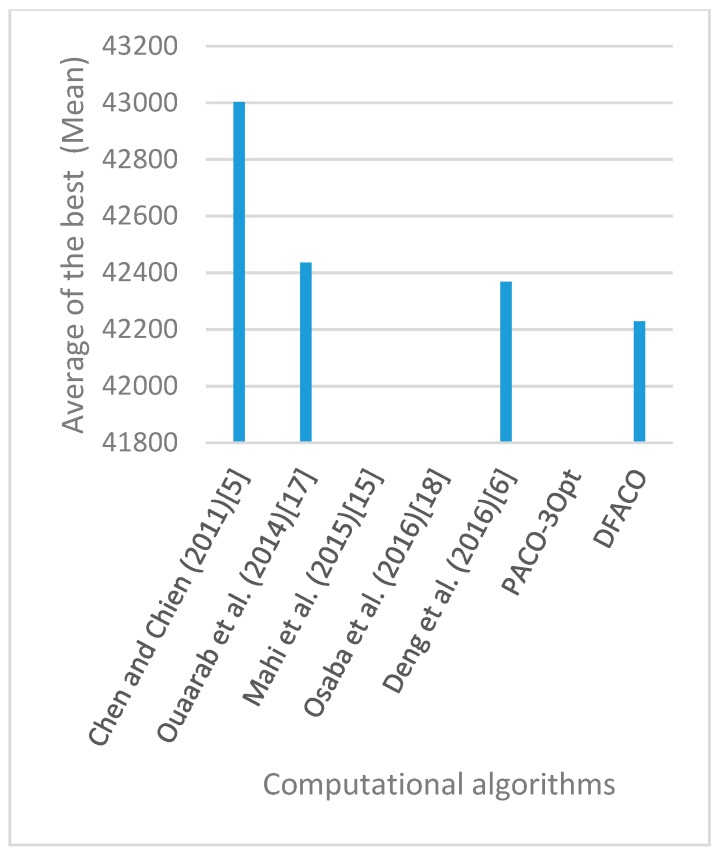
Average of best solutions obtained for instance lin318 by all algorithms.

**Figure 18:**
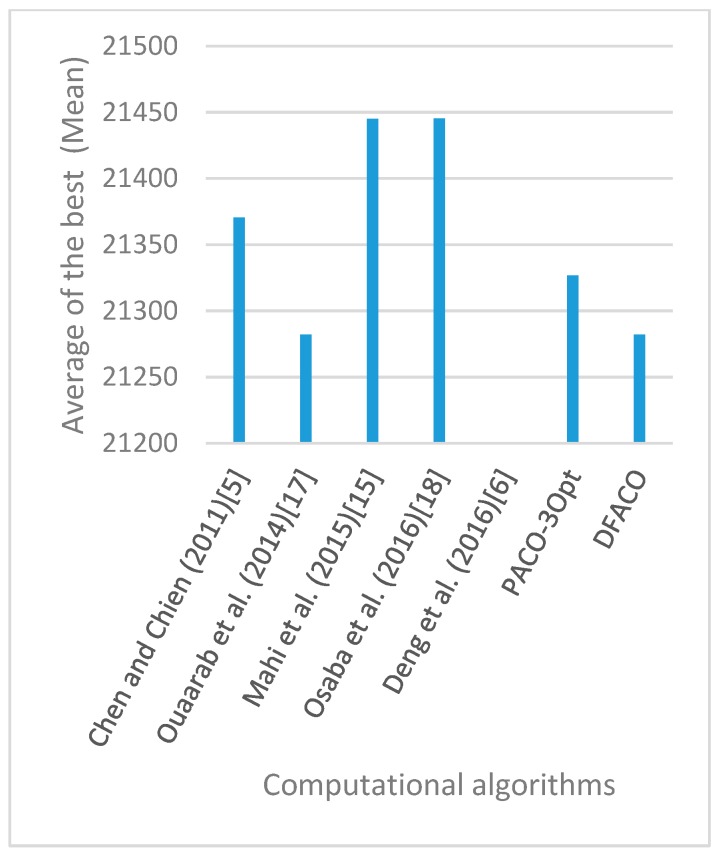
Average of best solutions obtained for instance kroA100 by all algorithms.

**Figure 19:**
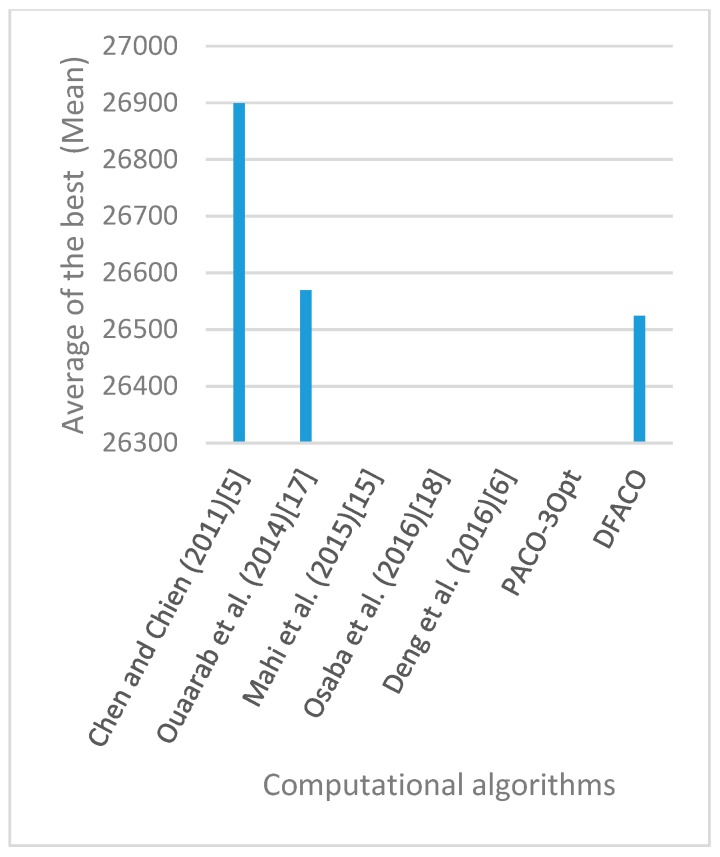
Average of best solutions obtained for instance kroA150 by all algorithms.

**Figure 20:**
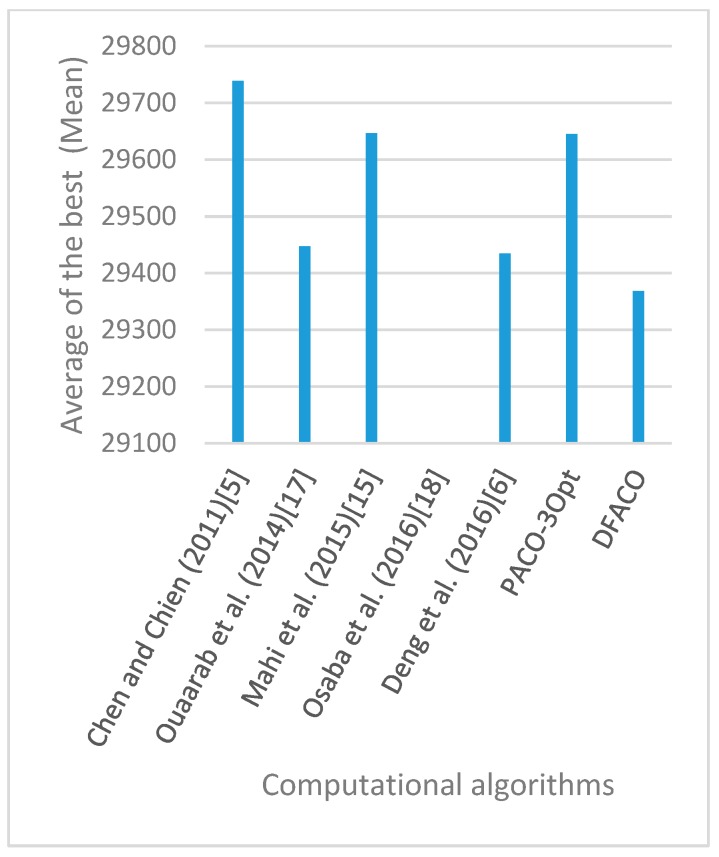
Average of best solutions obtained for instance kroA200 by all algorithms.

**Figure 21:**
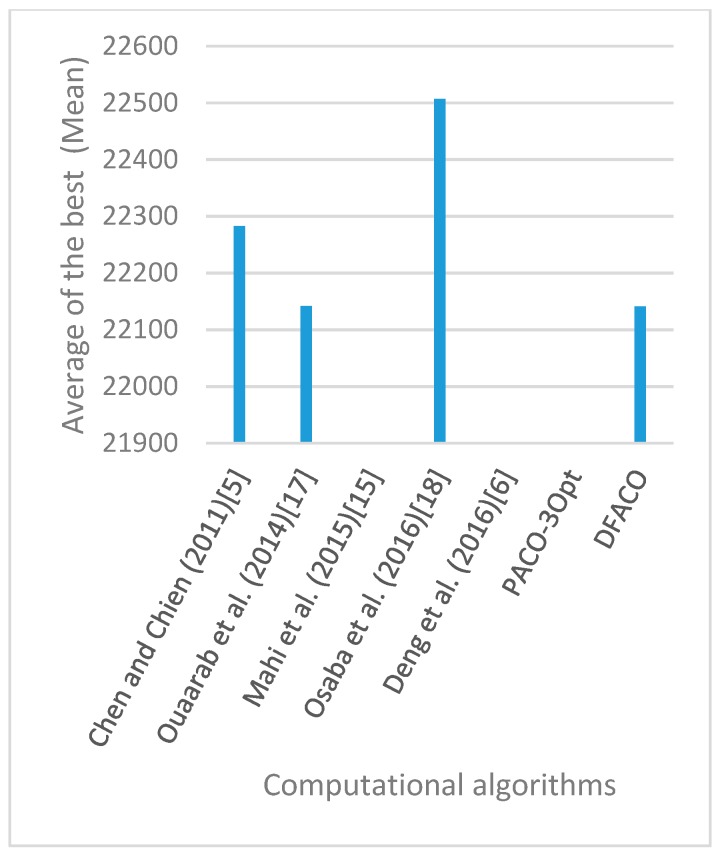
Average of best solutions obtained for instance kroB100 by all algorithms.

**Figure 22:**
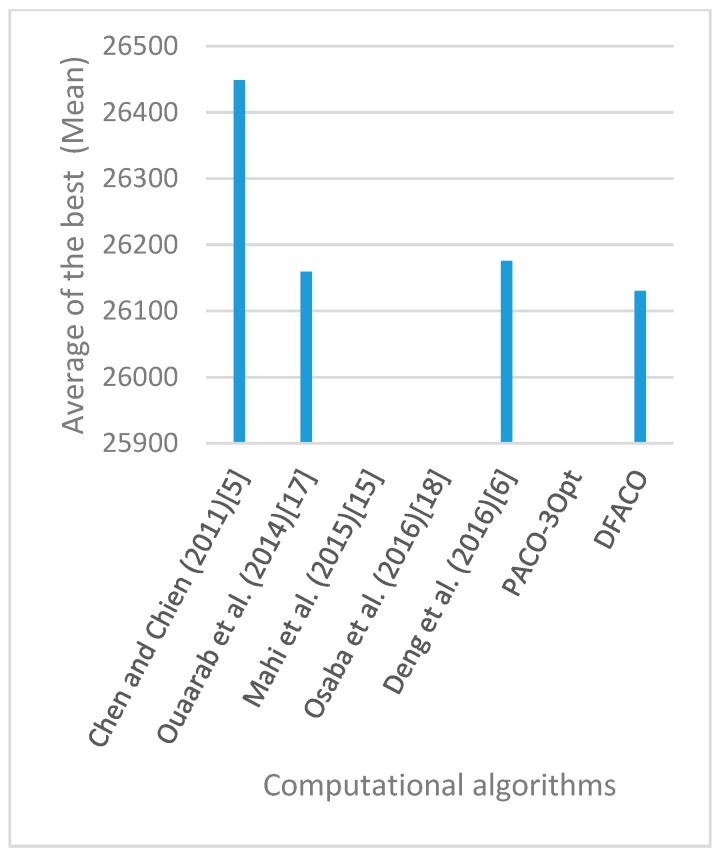
Average of best solutions obtained for instance kroB150 by all algorithms.

**Figure 23:**
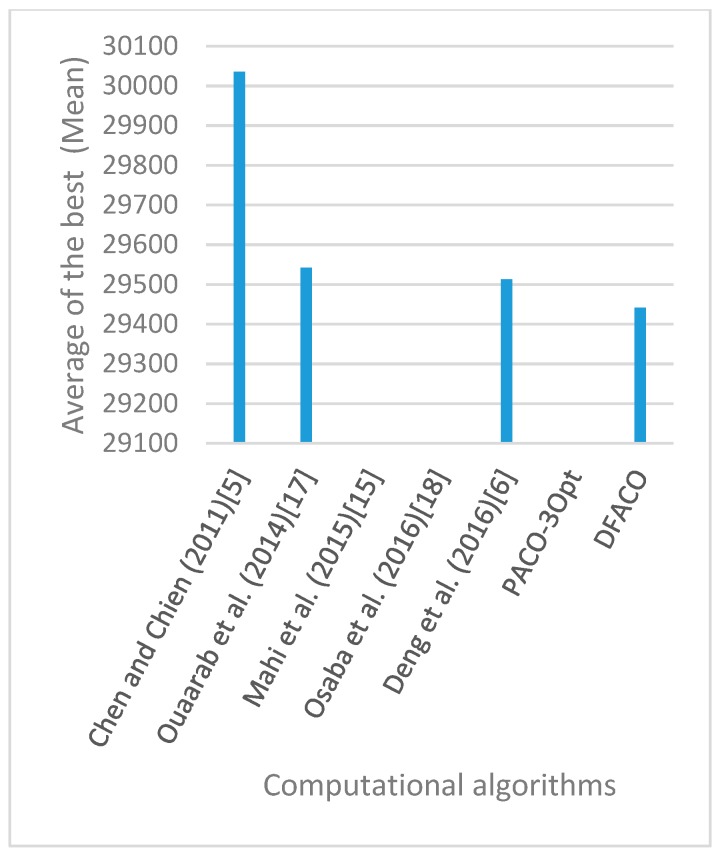
Average of best solutions obtained for instance kroB200 by all algorithms.

**Figure 24:**
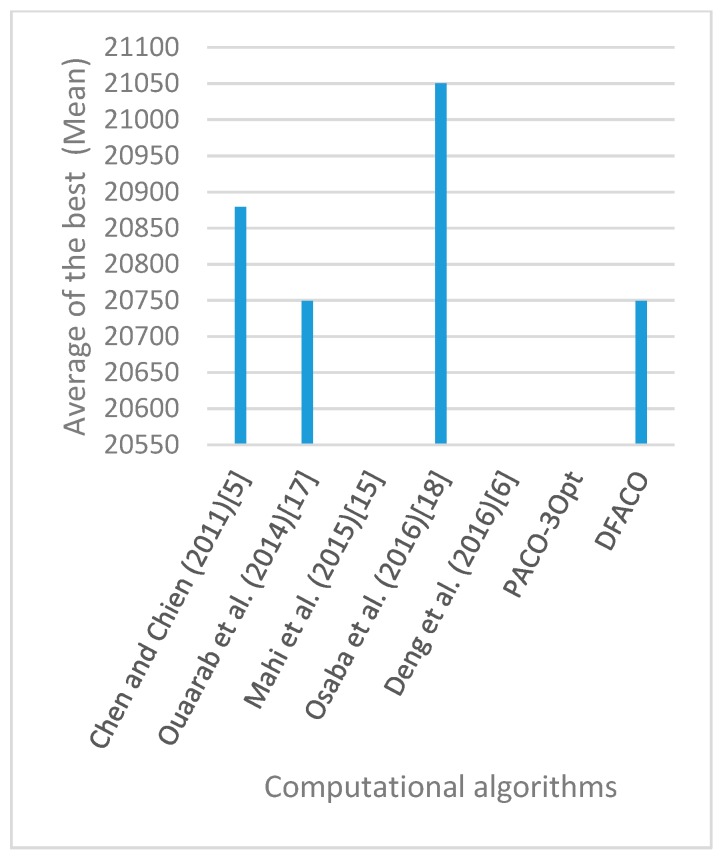
Average of best solutions obtained for instance kroC100 by all algorithms.

**Figure 25:**
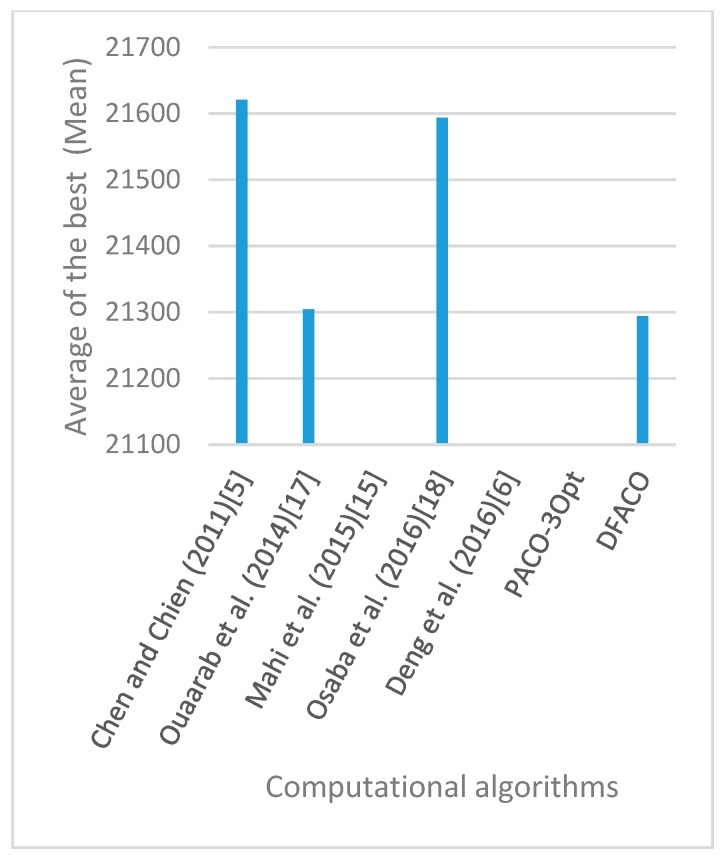
Average of best solutions obtained for instance kroD100 by all algorithms.

**Figure 26:**
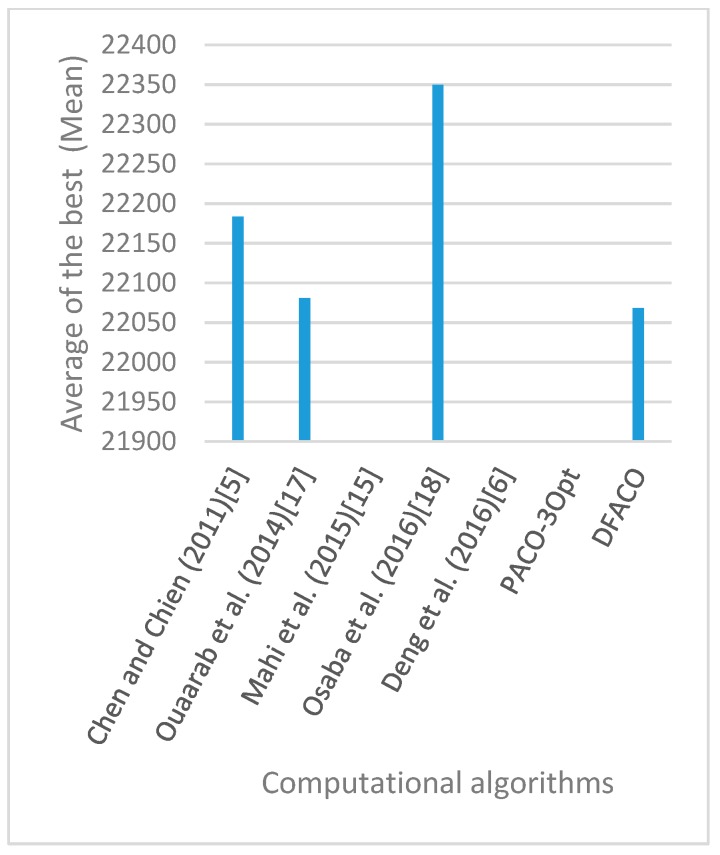
Average of best solutions obtained for instance kroE100 by all algorithms.

**Figure 27:**
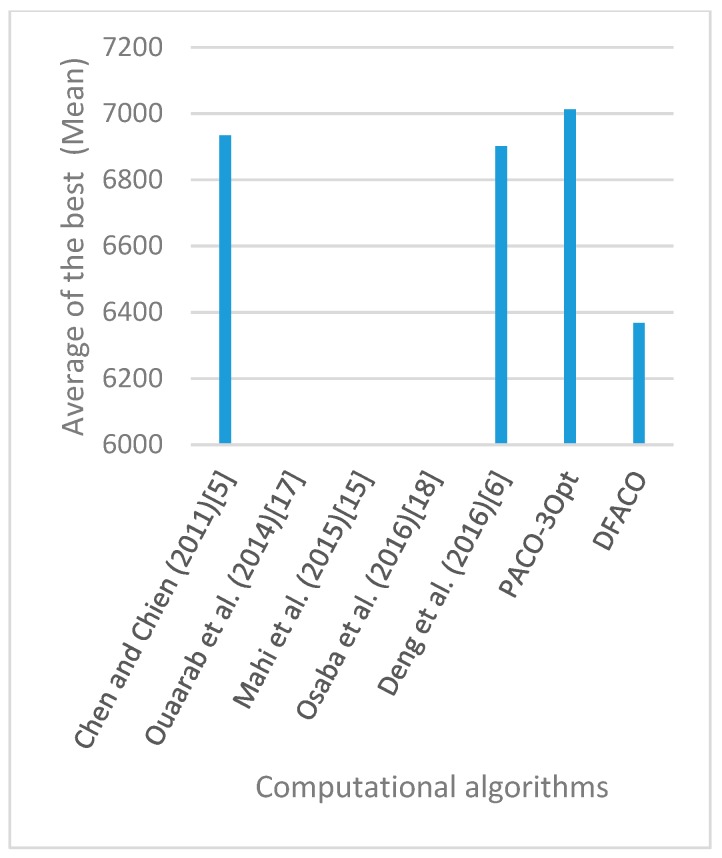
Average of best solutions obtained for instance rat575 by all algorithms.

**Figure 28:**
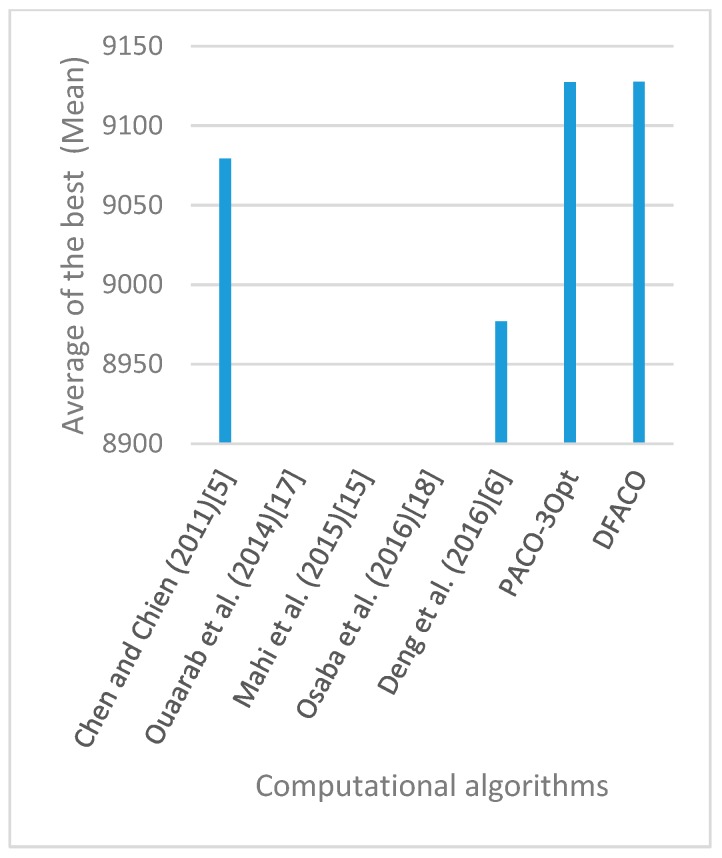
Average of best solutions obtained for instance rat783 by all algorithms.

**Figure 29:**
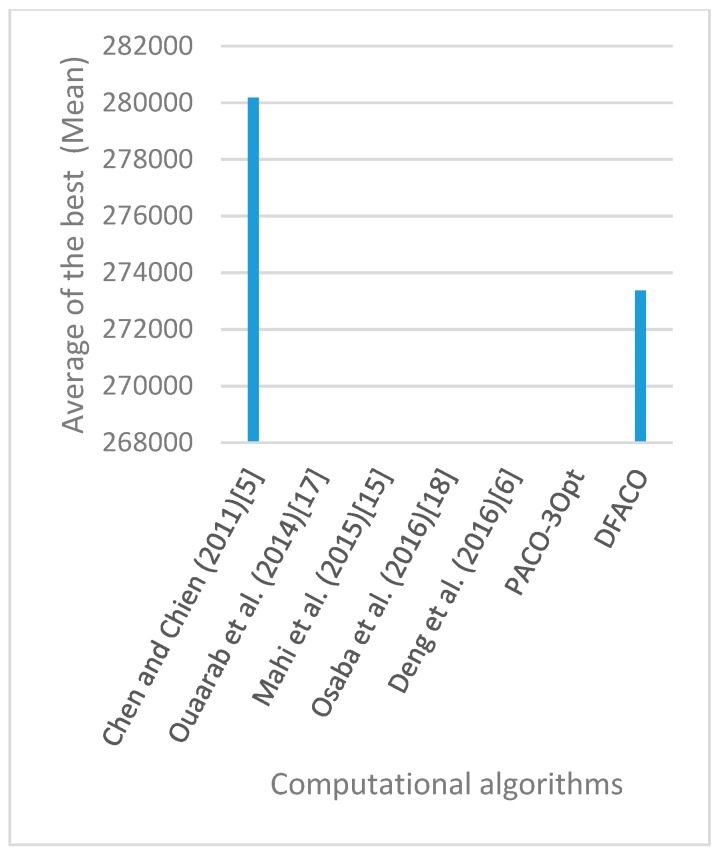
Average of best solutions obtained for instance rl1323 by all algorithms.

**Figure 30:**
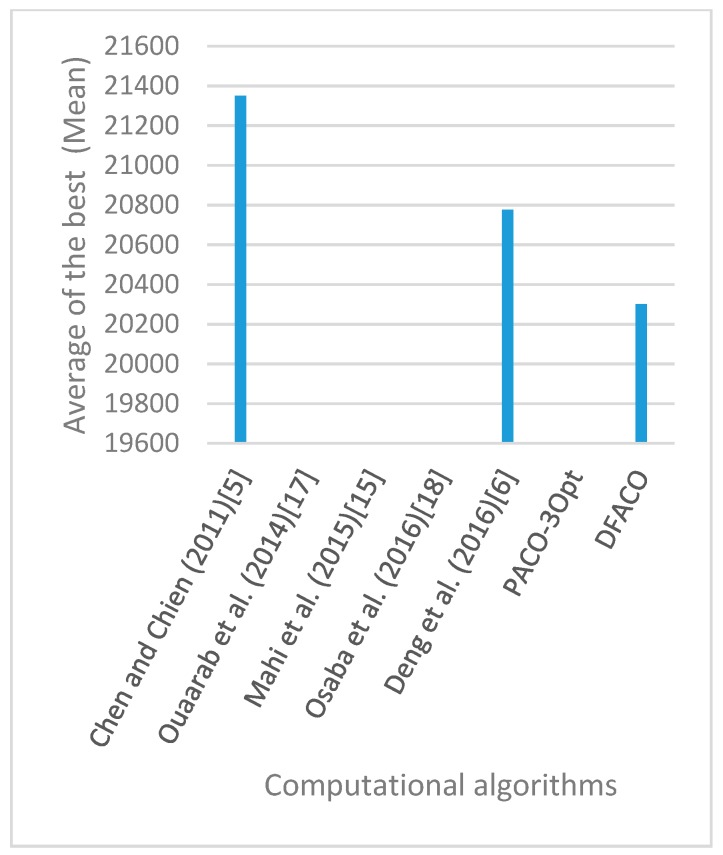
Average of best solutions obtained for instance fl1400 by all algorithms.

**Figure 31:**
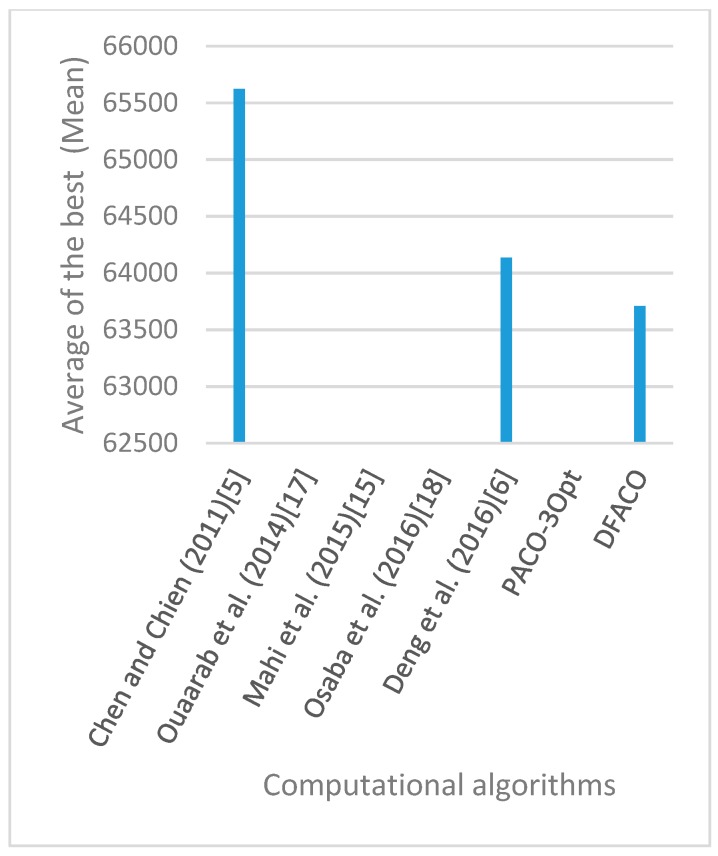
Average of best solutions obtained for instance d1655 by all algorithms.

**Table 1:** Control parameters for ACO and DFACO.

Parameters	Value
*α*	1
*β*	2
*ρ*	0.1
*τ*_0_	0.1
S	100
Z	100
Th (for DFACO)	80

**Table 2:** Experimental results of ACO and DFACO.

TSP Instance	BKS	ACO with 3-Opt				The Proposed Method			
		Mean	SD	Best	Time	Mean	SD	Best	Time
eil51	426	426.00	0.00	426.00	1	426.00	0.00	426.00	1
eil76	538	538.00	0.00	538.00	3	538.00	0.00	538.00	3
eil101	629	629.00	0.00	629.00	**10**	629.00	0.00	629.00	12
berlin52	7542	7542.00	0.00	7542.00	1	7542.00	0.00	7542.00	1
bier127	118,282	118,282.00	0.00	118,282.00	56	118,282.00	0.00	118,282.00	**47**
ch130	6110	6110.00	0.00	6110.00	16	6110.00	0.00	6110.00	**13**
ch150	6528	6528.00	0.00	6528.00	**17**	6528.00	0.00	6528.00	24
rd100	7910	7910.00	0.00	7910.00	2	7910.00	0.00	7910.00	2
lin105	14,379	14,379.00	0.00	14,379.00	2	14,379.00	0.00	14,379.00	2
lin318	42,029	42,243.70	52.27	42,135.00	**349**	**42,228.03**	48.30	**42,123.00**	381
kroA100	21,282	21,282.00	0.00	21,282.00	2	21,282.00	0.00	21,282.00	2
kroA150	26,524	26,524.07	0.25	26,524.00	83	**26,524.03**	0.18	26,524.00	**57**
kroA200	29,368	29,378.73	11.09	29,368.00	211	**29,368.00**	0.00	29,368.00	**168**
kroB100	22,141	22,141.00	0.00	22,141.00	2	22,141.00	0.00	22,141.00	2
kroB150	26,130	26,130.00	0.00	26,130.00	9	26,130.00	0.00	26,130.00	**7**
kroB200	29,437	29,443.20	7.07	29,437.00	**137**	**29,441.60**	5.16	29,437.00	185
kroC100	20,749	20,749.00	0.00	20,749.00	2	20,749.00	0.00	20,749.00	2
kroD100	21,294	21,294.00	0.00	21,294.00	3	21,294.00	0.00	21,294.00	3
kroE100	22,068	22,068.00	0.00	22,068.00	2	22,068.00	0.00	22,068.00	2
rat575	6773	6384.87	7.21	6368.00	443	**6367.3**	8.48	**6348.00**	**126.2**
rat783	8806	10,524.60	13.36	10,483.00	922	**10,491.9**	14.62	**10,455.00**	**151.6**
rl1323	270,199	273,969.57	515.57	272,639.00	**2267**	**273,367.9**	484.78	**272,487.00**	2285
fl1400	20,127	**20,291.77**	29.01	**20,225.00**	2471	20,300.77	35.27	20,239.00	**2452**
d1655	62,128	63,722.20	95.53	63,520.00	1754	**63,707.87**	114.84	**63,428.00**	**1523**

**Table 3:** DFACO and PACO-3Opt comparison on small-scale datasets.

TSP Instance	BKS	Best Tour Length		Worst Tour Length		Average Tour Length		Time (s)	
		DFACO	PACO-3Opt	DFACO	PACO-3Opt	DFACO	PACO-3Opt	DFACO	PACO-3Opt
Eil51	426	**426**	**426**	**426**	427	**426**	426.35	**1**	2.39
Berlin52	7542	**7542**	**7542**	**7542**	**7542**	**7542**	**7542**	**1**	2.1
Rat99	1211	**1211**	1213	**1211**	1225	**1211**	1217.1	**4**	19.79
Eil76	538	**538**	**538**	**538**	542	**538**	539.85	**3**	8.18
St70	675	**675**	676	**675**	679	**675**	677.85	**5.6**	6.97
KroA100	21,282	**21,282**	**21,282**	**21,282**	21,382	**21,282**	21,326.8	**2**	21.1
Lin105	14,379	**14,379**	**14,379**	**14,379**	14,422	**14,379**	14,393	**2**	14.57
KroA200	29,368	**29,368**	29,533	**29,368**	29,721	**29,368**	29,644.5	**168**	213.12
Ch150	6528	**6528**	6570	**6528**	6627	**6528**	6601.4	**24**	79.35
Eil101	629	**629**	**629**	**629**	639	**629**	630.55	**12**	20.79

**Table 4:** DFACO and PACO-3Opt comparison for large-scale datasets.

TSP Instance	BKS	Best Tour Length		Worst Tour Length		Average Tour Length		Time (s)	
		DFACO	PACO-3Opt	DFACO	PACO-3Opt	DFACO	PACO-3Opt	DFACO	PACO-3Opt
rd400	15,281.0	**15,321.0**	15,578.0	**15,428.0**	15,667.0	**15,384.0**	15,613.9	**133.1**	1496.0
fl417	11,861.0	**11,867.0**	11,972.0	**11,900.0**	12,000.0	**11,880.3**	11,987.4	**95.2**	2046.0
pr439	107,217.0	**107,310.0**	108,482.0	**107,698.0**	108,973.0	**107,515.9**	108,702.0	**142.6**	2132.0
pcb442	50,778.0	**50,910.0**	51,962.0	**51,186.0**	52,283.0	**51,047.4**	52,202.4	**129.4**	2088.0
d493	35,002.0	**35,124**	35,735.0	**35,380**	35,982.0	**35,266.4**	35,841.0	**137.59**	3175.0
u574	36,905.0	**37,168.0**	37,981.0	**37,518.0**	38,165.0	**37,366.8**	38,030.7	**116.9**	5460.0
rat575	6773.0	**6348.0**	7003.0	**6380.0**	7037.0	**6367.3**	7012.4	**126.2**	5004.0
p654	34,643.0	**34,695.0**	35,045.0	**34,864.0**	35,116.0	**34,740.6**	35,075.0	**101.7**	9105.0
d657	48,912.0	**49,250.0**	50,206.0	**49,618.0**	50,386.0	**49,462.7**	50,277.5	**135.1**	8715.0
u724	41,910.0	**42,284.0**	42,764.0	**42,634.0**	43,272.0	**42,437.8**	43,122.5	**137.0**	11,458.0
rat783	8806.0	10,455.0	**9111.0**	10,522.0	**9152.0**	10,491.9	**9127.3**	**151.6**	14,277.0

**Table 5:** Experimental results of proposed method compared with recent research (NA indicates that results were not reported in the original source).

TSP Instance	BKS	Chen and Chien (2011) [8]			Ouaarab et al. (2014) [20]			Mahi et al. (2015) [18]			Osaba et al. (2016) [21]			Deng et al. (2016) [9]			DFACO		
		Mean	SD	Best	Mean	SD	Best	Mean	SD	Best	Mean	SD	Best	Mean	SD	Best	Mean	SD	Best
eil51	426	427.27	0.45	427	426	0	426	426.45	0.61	426	428.1	1.6	426	427.21	NA	426	**426**	**0**	**426**
eil76	538	540.2	2.94	538	538.03	0.17	538	538.3	0.47	538	548.1	3.8	539	540.302	NA	539.153	**538**	**0**	**538**
eil101	629	635.23	3.59	630	630.43	1.14	629	632.7	2.12	631	646.4	4.9	634	634.68	NA	630.01	**629**	**0**	**629**
berlin52	7542	7542	0	7542	7542	0	7542	7543.2	2.37	7542	7542	0	7542	NA	NA	NA	**7542**	**0**	**7542**
bier127	118,282	119,421.8	580.83	118,282	118,360	12.73	118,282	NA	NA	NA	NA	NA	NA	NA	NA	NA	**118,282**	**0**	**118,282**
ch130	6110	6205.63	43.7	6141	6136	21.24	6110	NA	NA	NA	NA	NA	NA	6123.92	NA	6113.26	**6110**	**0**	**6110**
ch150	6528	6563.7	22.45	6528	6549.9	20.51	6528	6563.95	27.6	6538	NA	NA	NA	6539.86	NA	6528	**6528**	**0**	**6528**
rd100	7910	7987.57	62.06	7910	NA	NA	NA	NA	NA	NA	NA	NA	NA	7934.69	NA	7910	**7910**	**0**	**7910**
lin105	14,379	14,406.37	37.28	14,379	14,379	0	14,379	14,379.2	0.48	14,379	NA	NA	NA	14,394.1	NA	14,381.8	**14,379**	**0**	**14,379**
lin318	42,029	43,002.9	307.51	42,487	42,435	185.4	42,125	NA	NA	NA	NA	NA	NA	42,368.3	NA	42,284.9	**42,228.03**	**48.3**	**42,123**
kroA100	21,282	21,370.47	123.36	21,282	21,282	0	21,282	21,445.1	78.2	21,301	21,445.3	116.5	21,282	NA	NA	NA	**21,282**	**0**	**21,282**
kroA150	26,524	26,899.2	133.02	26,524	26,569	56.26	26,524	NA	NA	NA	NA	NA	NA	NA	NA	NA	**26,524.03**	**0.18**	**26,524**
kroA200	29,368	29,738.73	356.07	29,383	29,447	95.68	29,382	29,646.1	115	29,468	NA	NA	NA	29,434.7	NA	29,380.2	**29,368**	**0**	**29,368**
kroB100	22,141	22,282.87	183.99	22,141	22,142	2.87	22,141	NA	NA	NA	22,506.4	221.3	22,140	NA	NA	NA	**22,141**	**0**	**22,141**
kroB150	26,130	26,448.33	266.76	26,130	26,159	34.72	26,130	NA	NA	NA	NA	NA	NA	26,175.3	NA	26,139.4	**26,130**	**0**	**26,130**
kroB200	29,437	30,035.23	357.48	29,541	29,542	92.17	29,448	NA	NA	NA	NA	NA	NA	29,512.4	NA	29,502.6	**29,441.6**	**5.161**	**29,437**
kroC100	20,749	20,878.97	158.64	20,749	20,749	0	20,749	NA	NA	NA	21,050	164.7	20,749	NA	NA	NA	**20,749**	**0**	**20,749**
kroD100	21,294	21,620.47	226.6	21,309	21,304	21.79	21,294	NA	NA	NA	21,593.4	141.6	21,294	NA	NA	NA	**21,294**	**0**	**21,294**
kroE100	22,068	22,183.47	103.32	22,068	22,081	18.5	22,068	NA	NA	NA	22,349.6	169.6	22,068	NA	NA	NA	**22,068**	**0**	**22,068**
rat575	6773	6933.87	27.62	6891	NA	NA	NA	NA	NA	NA	NA	NA	NA	6901.25	NA	6859.85	**6367.3**	**8.48**	**6348**
rat783	8806	9079.23	52.69	8988	NA	NA	NA	NA	NA	NA	NA	NA	NA	**8976.92**	NA	**8940.37**	10,491.9	14.62	10,455
rl1323	270,199	280,181.5	1761.7	277,642	NA	NA	NA	NA	NA	NA	NA	NA	NA	NA	NA	NA	**273,367.9**	**484.8**	**272,487**
fl1400	20,127	21,349.63	527.88	20,593	NA	NA	NA	NA	NA	NA	NA	NA	NA	20,776.2	NA	20,683.8	**20,300.77**	**35.27**	**20,239**
d1655	62,128	65,621.13	1031.9	64,151	NA	NA	NA	NA	NA	NA	NA	NA	NA	64,133.7	NA	63,615.6	**63,707.87**	114.84	**63,428.00**

**Table 6:** Results of *PDav* and *PDbest* for DFACO compared with recent methods.

TSP Instance	BKS	Chen and Chien (2011) [8]		Ouaarab et al. (2014) [20]		Mahi et al. (2015) [18]		Osaba et al. (2016) [21]		Deng et al. (2016) [9]		DFACO	
		*PD_av_*	*PD_best_*	*PD_av_*	*PD_best_*	*PD_av_*	*PD_best_*	*PD_av_*	*PD_best_*	*PD_av_*	*PD_best_*	*PD_av_*	*PD_best_*
eil51	426	0.30	0.23	0.00	0.00	0.11	0.00	0.49	0.00	0.28	0.00	**0.00**	**0.00**
eil76	538	0.41	0.00	0.01	0.00	0.06	0.00	1.88	0.19	0.43	0.21	**0.00**	**0.00**
eil101	629	0.99	0.16	0.23	0.00	0.59	0.32	2.77	0.79	0.90	0.16	**0.00**	**0.00**
berlin52	7542	0.00	0.00	0.00	0.00	0.02	0.00	0.00	0.00	NA	NA	**0.00**	**0.00**
bier127	118,282	0.96	0.00	0.07	0.00	NA	NA	NA	NA	NA	NA	**0.00**	**0.00**
ch130	6110	1.57	0.51	0.42	0.00	NA	NA	NA	NA	0.23	0.05	**0.00**	**0.00**
ch150	6528	0.55	0.00	0.34	0.00	0.55	0.00	NA	NA	0.18	0.00	**0.00**	**0.00**
rd100	7910	0.98	0.00	NA	NA	NA	NA	NA	NA	0.31	0.00	**0.00**	**0.00**
lin105	14,379	0.19	0.00	0.00	0.00	0.00	0.00	NA	NA	0.11	0.02	**0.00**	**0.00**
lin318	42,029	2.32	1.09	0.97	0.23	NA	NA	NA	NA	0.81	0.61	**0.47**	**0.22**
kroA100	21,282	0.42	0.00	0.00	0.00	0.77	0.09	0.77	0.00	NA	NA	**0.00**	**0.00**
kroA150	26,524	1.41	0.00	0.17	0.00	NA	NA	NA	NA	NA	NA	**0.00**	**0.00**
kroA200	29,368	1.26	0.05	0.27	0.05	0.95	0.34	NA	NA	0.23	0.04	**d0.00**	**0.00**
kroB100	22,141	0.64	0.00	0.00	0.00	NA	NA	1.65	0.00	NA	NA	**0.00**	**0.00**
kroB150	26,130	1.22	0.00	0.11	0.00	NA	NA	NA	NA	0.17	0.04	**0.00**	**0.00**
kroB200	29,437	2.03	0.35	0.36	0.04	NA	NA	NA	NA	0.26	0.22	**0.02**	**0.00**
kroC100	20,749	0.63	0.00	0.00	0.00	NA	NA	1.45	0.00	NA	NA	**0.00**	**0.00**
kroD100	21,294	1.53	0.07	0.05	0.00	NA	NA	1.41	0.00	NA	NA	**0.00**	**0.00**
kroE100	22,068	0.52	0.00	0.06	0.00	NA	NA	1.28	0.00	NA	NA	**0.00**	**0.00**
rat575	6773	2.38	1.74	NA	NA	NA	NA	NA	NA	1.89	1.28	**−5.85**	**−6.19**
rat783	8806	3.10	2.07	NA	NA	NA	NA	NA	NA	1.94	1.53	19.30	19.01
rl1323	270,199	3.69	2.75	NA	NA	NA	NA	NA	NA	NA	NA	**1.17**	**0.85**
fl1400	20,127	6.07	2.32	NA	NA	NA	NA	NA	NA	3.23	2.77	**0.86**	**0.56**
d1655	62,128	5.62	3.26	NA	NA	NA	NA	NA	NA	3.23	2.39	**2.38**	**2.02**
